# EDIR: exome database of interspersed repeats

**DOI:** 10.1093/bioinformatics/btac771

**Published:** 2022-12-01

**Authors:** Laura D T Vo Ngoc, Randy Osei, Katrin Dohr, Catharina Olsen, Sara Seneca, Alexander Gheldof

**Affiliations:** Vrije Universiteit Brussel (VUB), Universitair Ziekenhuis Brussel (UZ Brussel), Clinical Sciences, Research Group Reproduction and Genetics, Centre for Medical Genetics, Brussels 1090, Belgium; Vrije Universiteit Brussel (VUB), Universitair Ziekenhuis Brussel (UZ Brussel), Clinical Sciences, Research Group Reproduction and Genetics, Centre for Medical Genetics, Brussels 1090, Belgium; Department of Paediatrics and Adolescent Medicine, Research Unit of Analytical Mass Spectrometry, Cell Biology and Biochemistry of Inborn Errors of Metabolism, Graz 8010, Austria; Vrije Universiteit Brussel (VUB), Universitair Ziekenhuis Brussel (UZ Brussel), Clinical Sciences, Research Group Reproduction and Genetics, Centre for Medical Genetics, Brussels 1090, Belgium; Brussels Interuniversity Genomics High Throughput Core (BRIGHTcore), VUB-ULB, Brussels 1090, Belgium; Interuniversity Institute of Bioinformatics in Brussels (IB)2, VUB-ULB, Brussels 1050, Belgium; Vrije Universiteit Brussel (VUB), Universitair Ziekenhuis Brussel (UZ Brussel), Clinical Sciences, Research Group Reproduction and Genetics, Centre for Medical Genetics, Brussels 1090, Belgium; Vrije Universiteit Brussel (VUB), Universitair Ziekenhuis Brussel (UZ Brussel), Clinical Sciences, Research Group Reproduction and Genetics, Centre for Medical Genetics, Brussels 1090, Belgium

## Abstract

**Motivation:**

Intragenic exonic deletions are known to contribute to genetic diseases and are often flanked by regions of homology.

**Results:**

In order to get a more clear view of these interspersed repeats encompassing a coding sequence, we have developed EDIR (Exome Database of Interspersed Repeats) which contains the positions of these structures within the human exome. EDIR has been calculated by an inductive strategy, rather than by a brute force approach and can be queried through an R/Bioconductor package or a web interface allowing the per-gene rapid extraction of homology-flanked sequences throughout the exome.

**Availability and implementation:**

The code used to compile EDIR can be found at https://github.com/lauravongoc/EDIR. The full dataset of EDIR can be queried via an Rshiny application at http://193.70.34.71:3857/edir/. The R package for querying EDIR is called ‘EDIRquery’ and is available on Bioconductor. The full EDIR dataset can be downloaded from https://osf.io/m3gvx/ or http://193.70.34.71/EDIR.tar.gz.

**Supplementary information:**

Supplementary data are available at *Bioinformatics* online.

## 1 Introduction

Intragenic exonic deletions have been shown to contribute to genetic defects in a wide subset of genes and diseases (Alessandri *et al.*, 2018; Azuma *et al*., 2017; Brett *et al.*, 2017; Cai *et al.*, 2021; Chen *et al.*, 2019; Cotta *et al.*, 2020; Darin *et al.*, 2018; Duployez *et al.*, 2019; Esposito *et al.*, 2017; Grieco *et al.*, 2018; Guarnieri *et al.*, 2017; Iwata-Otsubo *et al.*, 2018; Keegan *et al.*, 2019; Knight Johnson *et al.*, 2017; Li *et al.*, 2020; Romaniello *et al.*, 2021; Uchiyama *et al.*, 2020; Wei *et al.*, 2021; Xie *et al.*, 2017). While many of these deletions have been identified to date, it may be assumed that their contribution to genetic diseases has been underestimated as they might have been missed by standard diagnostic next-generation sequencing (NGS) and micro-array methodologies. Possible mechanisms for these deletions are proposed to be recombination or replication based, as the removed sequences are often flanked by regions of homology. Furthermore, non-recurrent rearrangements are associated with shorter regions of microhomology of 70 bp or less (Carvalho and Lupski, 2016; Ottaviani *et al.*, 2014; Vissers *et al.*, 2009).

Our group has recently identified a Pompe’s disease patient who is compound heterozygous for the NM_000152.4(GAA): c.2331 + 2T>A, p.? alteration together with the 343 bp NM_000152.4(GAA): c.1327-61_1437 + 172del, p.? deletion spanning exon 9 and which is flanked by a 7 bp CCCCGTG repeat. This hints to the presence of microhomology as the major prerequisite for the deletion. Using the UCSC repeat browser tool (https://repeatbrowser.ucsc.edu/, accessed on January 20, 2022), we were unable to identify the presence of known repetitive elements in the breakpoint region of the above-detected deletion (Fernandes *et al.*, 2020). Furthermore, using RepeatMasker (v 4.0.9), RepeatScout (v 1.0.5) and the msRepDB database (https://msrepdb.cbrc.kaust.edu.sa/—accessed on May 11, 2022) we could also not identify the CCCCGTG interspersed repeat which was detected (Supplementary File S1). This points to an unmet need for a repository of this kind of interspersed repeats which are not connected to known transposable elements. In order to get a clearer view on the presence of similar structures, we have constructed a precompiled database called EDIR (Exome Database of Interspersed Repeats) which contains the positions of these structures within the exome.

## 2 Materials and methods

Compilation of EDIR was accomplished in four distinct steps: (i) mapping of interspersed repeat sequence (IRS) structures starting with a 7 bp repeat seed sequence (written in Python) and (ii) annotation and selection of the mapped structures according to their position in the exome (written in R), followed by (iii) extension of the 7 bp repeat to 20 bp. An additional user-friendly script was written (iv) allowing querying of the data base through a web interface (using R Shiny). EDIR was furthermore calculated allowing a 1 bp mismatch between any two flanking repeat sequences.

**Fig. 1.**
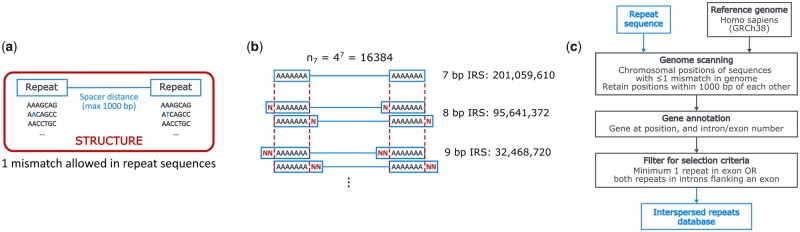
(**a**) Graphical depiction of an interspersed repeat structure (IRS) which is a spacer sequence up to 1000 bp flanked by two repeats. (**b**) Inductive strategy to identify IRS’s with repeats >7 bp. The set of IRS with all possible 7 bp sequences also contains the set of IRS with 8 bp repeats, while the 8 bp set will in fact contain the 9 bp set and so on. As the possible number of sequence combinations increases by a power of four when the repeat is extended by 1 bp, this subsetting methodology makes it possible to identify IRS’s containing long repeat sizes which would otherwise be impractical or virtually impossible to compute due to the large number of combinations. (**c**) General overview of the used methodology to compile the EDIR database for 7 bp repeat IRS’s

We define an IRS as a sequence of maximum of 1000 bp flanked by two repeats with are identical or have a mismatch of maximum of 1 bp (Fig. 1a). We started by scanning the human genome (GRCh38 or Hg38) for all permutations of 7 bp sequences (4^7^ = 16 384) and once mapped, the genomic start and stop positions of IRS were stored. We aimed to include only IRS in which minimally one repeat was located in an exon or both repeats were situated in different introns flanking one or more exons. As such, a selection of regions has been done which, in case of recombination or replication errors, could potentially result in the expression of aberrant mRNA and protein and thus could lead to disease. For this purpose, the start and end positions of all detected 7 bp IRS were compared to the start and stop positions of all known exons. (UCSC-knownCanonicalTranscripts: http://hgdownload.soe.ucsc.edu/ goldenPath/hg38/database/File date: October 13, 2019). The calculation (mapping and intron-exon selection) for all 7 bp permutations allowing a maximum of one mismatch was performed on a virtual machine with 40 cores and 40 Gb of RAM and took ±193 h to complete and resulted in a 60 Gb data file. As we aimed to extend our analysis towards 8–20 bp repeats, the number of possible permutations of for instance the 20 bp IRS even without a 1 bp mismatch would attain 4^20^ > 1E12, a combination size too large for calculation. However, theoretically, the generated data of the 7 bp IRS also encompasses the sets of 8 bp and larger (Fig. 1b). Identification of the 8 bp IRS set was done by extending the previously mapped 7 bp repeats with 1 bp increments. If two 7 + 1 bp repeats mapped as an IRS, they were kept in the 8 bp dataset and each time a mismatch was encountered, the specific IRS was not extended anymore unless the expanded bases were identical. This process was iterated until a repeat length of 20 bp was reached, while always maintaining a maximum string distance between both repeats in the IRS of 1 bp. A general overview of the methodology used can be found in Figure 1c.

This has resulted in an R package which is available on Bioconductor and which contains the different IRS for the *GAA* gene. The complete EDIR dataset can be downloaded at: https://osf.io/m3gvx/ and http://193.70.34.71/EDIR.tar.gz (direct download) and the location of the files provided to the query function is https://github.com/lauravongoc/EDIR.

## 3 Results

An overview of the general EDIR data is depicted in Supplementary Figure S1. Chromosome 19 harbors the largest amount of genes with at least one 20 bp IRS (Supplementary Fig. S1a). While it is known that chromosome 19 is the most gene dense, nevertheless, a smaller total amount of genes is found in comparison to several chromosomes including one and two. The largest number of 20 bp IRS per gene can be observed in chromosomes 15, 16 and 19 with respectively 62, 58 and 65k instances. Interestingly, while approximately 20 million IRS with a 7 bp repeat exist within the known exome, this dramatically decreases to nearly 500 000 instances in the case of 20 bp repeats (Supplementary Fig. S1b). We created an R-based script which is able to query the generated database and is available on Bioconductor as ‘EDIRquery’. By using the ‘gene_lookup’ function, EDIR can be queried for IRS in a gene of interest. Additional parameters which can be entered are the length of the repeat (ranging from 7 to 20 bp), the minimum (≥0 bp) and maximum distance (≤1000 bp) of the spacer sequence, and whether to allow a 1-bp mismatch. As output, a table is given where for each repeat length, the number of interspersed repeat structures, together with the average distance separating two repeats, as well as the number of interspersed repeat structures per megabase and whether a 1 bp mismatch has occurred. This general data can be extended towards a more detailed overview depicting the genomic start and stop positions of each IRS by assigning the output of the ‘gene_lookup’ function to a variable which can be interrogated by standard R practices. For ease of use, this functionality has been embedded in a web interface which is accessible at http://193.70.34.71:3857/edir/ in which the queried data is downloadable in a .csv format. In addition, the web interface links each IRS to its genomic region in the UCSC Genome Browser (https://genome.ucsc.edu), ClinVar (https://www.ncbi.nlm.nih.gov/clin-var) and dbVar (https://www.ncbi.nlm.nih.gov/dbvar/).

## 4 Discussion

We developed a repository for interspersed repeats located within the exome, which has been created by means of an inductive strategy and which is not based on a priori knowledge of known transposable elements (TEs). Nevertheless, our approach to IRS identification still allows for the inclusion of these known TEs, while most importantly not being limited to them. For instance, our initially detected CCCCGTG repeat flanking exon 9 in the *GAA* gene cannot be attributed to any known TE-like element in the msRepDB repository, while several IRS containing this repeat can indeed be traced to TEs outside the GAA gene (Liao *et al.*, 2022).

With this broad scope, EDIR can amongst others be used to couple exonic deletions to certain IRS’s and vice versa, to identify which IRS’s and/or genomic regions are more prone to recombinatory effects. Furthermore, EDIR may guide genetic researchers to investigate specific exons for deletions in case of patients where only a single pathogenic alteration has been found in a gene with recessive inheritance. Because the collection of IRS’s is wider than known TE-related sequences this can also be done at a finer scale.

We have constructed EDIR by making use of an inductive algorithm whereby we reasoned that each set of IRS with repeats of *n* bp in length naturally contains within it the set of IRS with repeats of *n* + 1 bp in length. By applying this methodology, calculating IRS with large repeats (e.g. larger than 100) is possible.

## Supplementary Material

btac771_Supplementary_Data

## Data Availability

All data are incorporated into the article and its online supplementary material.
