# Supplementary material for: EDIR: exome database of interspersed repeats
Source: Bioinformatics. 2022 Dec 1;39(1):btac771. doi: 10.1093/bioinformatics/btac771 (PMC9805566; doi:10.1093/bioinformatics/btac771)
Supplement: btac771_Supplementary_Data [file btac771_supplementary_data.zip › btac771_Supplementary_Data/supplementary Fig 1.docx]

**Supplementary Fig. 1:** (**a**) Number of genes per chromosome containing at least one IRS flanked by 20 bp repeats (bars) together with the total number of genes located on each chromosome (line). (**b**) Overview of the total amount of IRS with different repeat sizes contained within EDIR. (**c**) Total amount IRS with 20 bp repeats for each chromosome. (**d**) The normalized number of IRS with repeats ranging from 7-20bp for each gene.
