# Supplementary material for: EDIR: exome database of interspersed repeats
Source: Bioinformatics. 2022 Dec 1;39(1):btac771. doi: 10.1093/bioinformatics/btac771 (PMC9805566; doi:10.1093/bioinformatics/btac771)
Supplement: btac771_Supplementary_Data [file btac771_supplementary_data.zip › btac771_Supplementary_Data/supplementary File 1.docx]

**Overview of repetitive sequences detected in the genomic region of exon 9 of the GAA gene with different repeat detection tools.**

Legend:

Exon 9 of the Homo sapiens GAA gene highlighted in green. CCCCGTG repeat highlighted in yellow. Masked based depicted as N are associated with repetitive sequences. The input sequence begins with the start of exon 8 of the *GAA* gene and ends with the last base of exon 10. This region thus encompasses the deletion of exon 9 we have originally detected. Genomic sequence used: NG_009822.1

1. Output of RepeatMasker (v 4.0.9)

>hsGAAeX8_10

GACGTCCAGTGGAACGACCTGGACTACATGGACTCCCGGAGGGACTTCAC

GTTCAACAAGGATGGCTTCCGGGACTTCCCGGCCATGGTGCAGGAGCTGC

ACCAGGGCGGCCGGCGCTACATGATGATCGTGGTGTGTGCCCCCACACTG

TGGGTCTTTGGGAAGGGGGCCGCCCGGTGCCCAGTGGCTCCTTCTCTGTG

CAGCGTCATCCTCGTGCCTGTGTGGTCGCCGAGGATGTTTTCTGAGGGTC

TTTGTGATATCGAGGGAATATCAAGAAGTTTGCAGGCTTGGCCCCAGCTG

TCCAGGGAGGTCGGGTTTGAGGGTCCCCAGAAATGGCCGGGTGCNNNNNN

NNNNNNNNNNNNNNNNNNNNNNNNNNNNNNNNNNNNNNNNNNNNNNNNNN

NNNNNNNNNNNNNNNNNNNNNNNNNNNNNNNNNNNNNNNNNNNNNNNNNN

NNNNNNNNNNNNNNNNNNNNNNNNNNNNNNNNNNNNNNNNNNNNNNNNNN

NNNNNNNNNNNNNNNNNNNNNNNNNNNNNNNNNNNNNNNNNNNNNNNNNN

NNNNNNNNNNNNNNNNNNNNNNNNNNNNNNNNNNNNNNNNNNNNNNNNNN

NNNNNNNNNNNNNNNNNNNNNNNNNNNNNNNNNNNNNNNNNNNNNNNNNN

NNNNNNNNNNNNNNNNNNNNNNNNNNNNNNNNNNNNNNNNNNNNNNNNNN

NNNNNNNNNNNNNNNNNNNNNNNNNNNNNNNNNNGGCACGATGGCCAGAG

GAGGAGGTGGGAGGCAGGGCGAGCTGAAAAGATCCAACAGTTCCTGCCCG

GAAGATCCACTTCAGCAGAGGAAGCACAGATGAGATGTGGGGCTGTGCTG

ATGCTGCCTGTTTCCATCCCTGCCTTCTGCAGGCAGCAAACAGTAGTAGC

CCTTAAGAGCAGGAGTGGAAACACAGACTTTTTTCTTTCTCACANNNNNN

NNNNNNNNNNNNNNNNNNNNNNNNNNNNNNNNNNNNNNNNNNNNNNNNNN

NNNNNNNNNNNNNNNNNNNNNNNNNNTTCCATTCCGGCGCGCCCCTCATC

AGCCAGCTGGTCCTGACTCGCCCGGCCCTGGCTCCTCTCNNNNNNNNNNN

NNNNNNNNNNNNNNNNNNNNNNNNNNNNNNNNNNTGTACACACGCATGAT

GTCATCCCCAGCCTCATCCTCTCACTGTCTCAGTTTT**CCCCGTG**GCTGGC

GCCAGGGCTCTGGGCCACCCTCACCTTGACAGGTTTCCCTCTTCCCAGGA

TCCTGCCATCAGCAGCTCGGGCCCTGCCGGGAGCTACAGGCCCTACGACG

AGGGTCTGCGGAGGGGGGTTTTCATCACCAACGAGACCGGCCAGCCGCTG

ATTGGGAAGGTAGGGCGAGGGTCCAGGGGACGGGGGTTAGAAAGCAGAGG

CCTCCAGCCAGGGGGAGCCGGCAGCTGCTCAGGAAGACGGTGGGATTTGA

GGAGCCATCACGCCCAGTGGGACAGCTGAGAGGAATGGGCCACAGTGGCC

CGTGACGATGGTGGCTCCTACAAGGAATGG**CCCCGTG**AGTTCTTCCATCA

GCAGGCCTTTGACTTCATGGGCAGCTGGGCCTGGCCCAGGCACAAGCCCT

GCAGACCCTCAGTGAGGCCTTAGGGTCCTCCTTGTCCTCCCAGCCCCCCA

GGGGCCTCCAGGCAGGGCCCCCGCTGAGGGAGCAGCTAGGGAGGGTCTGG

TGCGGATGTGAGGCTGCCTGGCAGGGCTTGCACGGGGCCGTCTCCGCTGC

CCTTCTCCCTGACGCTCTCTGGTTCTGCAGCCCAGCCCCTGGGTGGACGT

GTTGGGGGTGACCCCTCGTTTTCCCAGGGTTGAGGCCCCTTGGCCCCGCA

TCAGTGCCTTGTGGAGAAAGAGCTGCTCATTGACCTCCAGGGTGCAGGTC

TCTCAGATTTGCAAATGTGGGCGTCCACTAAGAGTGAGGCTGCCCCTCTG

CTCAGGCTGAGGCTCAGTGGGGCTTCCATGCAGGCCCTGGGTGGGGCCGG

GTCTCCCCACTGCAGCCTCTCGTTGTCCAGGTATGGCCCGGGTCCACTGC

CTTCCCCGACTTCACCAACCCCACAGCCCTGGCCTGGTGGGAGGACATGG

TGGCTGAGTTCCATGACCAGGTGCCCTTCGACGGCATGTGGATT

1. Output of msRepDB ((<https://msrepdb.cbrc.kaust.edu.sa/> - accessed on 11 May 2022)

>hsGAAeX8_10

GACGTCCAGTGGAACGACCTGGACTACATGGACTCCCGGAGGGACTTCAC

GTTCAACAAGGATGGCTTCCGGGACTTCCCGGCCATGGTGCAGGAGCTGC

ACCAGGGCGGCCGGCGCTACATGATGATCGTGGTGTGTGCCCCCACACTG

TGGGTCTTTGGGAAGGGGGCCGCCCGGTGCCCAGTGGCTCCTTCTCTGTG

CAGCGTCATCCTCGTGCCTGTGTGGTCGCCGAGGATGTTTTCTGAGGGTC

TTTGTGATATCGAGGGAATATCAAGAAGTTTGCAGGCTTGGCCCCAGCTG

TCCAGGGAGGTCGGGTTTGAGGGTCCCCAGAAATGGCCGGGTGCTACTCA

GGGTTCTGTCAGATGTAGGTTACTTGAACTGCCTTAAAGCAAAAGGCCAG

GGGCATGATAAACTGATGTCACCTGGTCCTGGAAAGTGGAGGGCCCGGTG

GGCCTGGGCATGGGTATCGCTGGAACTGTGGAGGCTCCGTGTGCCTTCTG

GCCGTGCCTCTCCTTCTGGCCGGCTCTGAATCCCTGGAAAGGACGGCGTG

AGTGAGGGCAGCTTCCAGCCCTCATGCTGGCACCACAGAGCGGAGACTTC

TTCCCATCAGCTCCCATAGAAAAGTCCCAAAGCAGGACTCTTGAGTCACC

CAGCACAAAGAGGCCCTTCCCTGAGCCAGTCCCACAGCCAGAAGGATGCA

GTTTGGGGGCTGGTCCAGCCCGAGTCTGGTGTCCGGCACGATGGCCAGAG

GAGGAGGTGGGAGGCAGGGCGAGCTGAAAAGATCCAACAGTTCCTGCCCG

GAAGATCCACTTCAGCAGAGGAAGCACAGATGAGATGTGGGGCTGTGCTG

ATGCTGCCTGTTTCCATCCCTGCCTTCTGCAGGCAGCAAACAGTAGTAGC

CCTTAAGAGCAGGAGTGGAAACACAGACTTTTTTCTTTCTCACATTTTTT

TAATTATAAAAGAAAAGTGATTACTGTAGAACACTTGGGAAACTCTAGAG

GTTTAAAGAAAAGGTAAAGGTAAAGCTTCCATTCCGGCGCGCCCCTCATC

AGCCAGCTGGTCCTGACTCGCCCGGCCCTGGCTCCTCTCNNNNNNNNNNN

NNNNNNNNNNNNNNNNNNNNNNNNNNNNNNNNNNTGTACACACGCATGAT

GTCATCCCCAGCCTCATCCTCTCACTGTCTCAGTTTT**CCCCGTG**GCTGGC

GCCAGGGCTCTGGGCCACCCTCACCTTGACAGGTTTCCCTCTTCCCAGGA

TCCTGCCATCAGCAGCTCGGGCCCTGCCGGGAGCTACAGGCCCTACGACG

AGGGTCTGCGGAGGGGGGTTTTCATCACCAACGAGACCGGCCAGCCGCTG

ATTGGGAAGGTAGGGCGAGGGTCCAGGGGACGGGGGTTAGAAAGCAGAGG

CCTCCAGCCAGGGGGAGCCGGCAGCTGCTCAGGAAGACGGTGGGATTTGA

GGAGCCATCACGCCCAGTGGGACAGCTGAGAGGAATGGGCCACAGTGGCC

CGTGACGATGGTGGCTCCTACAAGGAATGG**CCCCGTG**AGTTCTTCCATCA

GCAGGCCTTTGACTTCATGGGCAGCTGGGCCTGGCCCAGGCACAAGCCCT

GCAGACCCTCAGTGAGGCCTTAGGGTCCTCCTTGTCCTCCCAGCCCCCCA

GGGGCCTCCAGGCAGGGCCCCCGCTGAGGGAGCAGCTAGGGAGGGTCTGG

TGCGGATGTGAGGCTGCCTGGCAGGGCTTGCACGGGGCCGTCTCCGCTGC

CCTTCTCCCTGACGCTCTCTGGTTCTGCAGCCCAGCCCCTGGGTGGACGT

GTTGGGGGTGACCCCTCGTTTTCCCAGGGTTGAGGCCCCTTGGCCCCGCA

TCAGTGCCTTGTGGAGAAAGAGCTGCTCATTGACCTCCAGGGTGCAGGTC

TCTCAGATTTGCAAATGTGGGCGTCCACTAAGAGTGAGGCTGCCCCTCTG

CTCAGGCTGAGGCTCAGTGGGGCTTCCATGCAGGCCCTGGGTGGGGCCGG

GTCTCCCCACTGCAGCCTCTCGTTGTCCAGGTATGGCCCGGGTCCACTGC

CTTCCCCGACTTCACCAACCCCACAGCCCTGGCCTGGTGGGAGGACATGG

TGGCTGAGTTCCATGACCAGGTGCCCTTCGACGGCATGTGGATT

1. Output of RepeatScout (v 1.0.5)

The CCCCGTG repeat sequence is not represented in the l-mer frequency table below (min. l-mers: 2).

>hsGAAeX8_10

ACCAGGT 2 11420

AAGAAGT 2 11272

AGCAGCT 2 12680

ACTGCCT 2 13045

AGGCCCT 3 12981

AAGCCCT 2 12593

AGACCCT 3 12691

AAATGTG 2 11940

CACAGTG 2 11145

AGGGCTG 2 13073

ACAGCTG 2 11294

AGCCCTG 3 12721

AACCCTG 2 12824

ACTGATG 2 11411

AAGGATG 2 11691

CATCATG 2 12144

CAGCATG 2 12129

CACCATG 2 13095

ACTGTGG 2 11474

ATGGTGG 2 13096

CAGCTGG 4 12052

CACCTGG 2 11419

CCACTGG 2 12463

ACGATGG 2 12504

ATGTGGG 2 12914

CAGTGGG 3 12964

AGCTGGG 2 11292

CACTGGG 2 12462

AGTGGGG 2 12965

CACGGGG 2 12530

CCAGGGG 3 12647

ACGAGGG 2 12297

CCCAGGG 2 12822

AGTGAGG 2 12932

AGGGAGG 2 12687

AGGCAGG 3 12659

AGCCAGG 2 12076

CACCAGG 2 11099

ACACAGG 2 11216

CAGAAGG 2 11872

CAGCTCG 2 11769

ATCCTCG 2 11207

CCGGGCG 2 12068

CAGGGCG 2 11764

CCCTCCG 2 12308

ATGGCCG 2 11079

CCCACCG 2 12437

ACTCACG 2 12533

ATGTGAG 2 11938

CAGTGAG 2 12170

CCCTGAG 2 11346

CCTGGAG 2 12884

CCGGGAG 2 11032

CAGGGAG 2 11302

AGCGGAG 2 11588

AGAGGAG 2 11746

CAACGAG 2 12328

AGCAGAG 3 12392

CACAGAG 2 11193

CTCTCAG 2 12475

CCCTCAG 3 12673

CCATCAG 2 12544

CCTGCAG 2 11876

AGGGCAG 2 11554

CCGGCAG 2 12273

CAGGCAG 4 12713

AAGGCAG 2 13046

AAAGCAG 2 12390

CGGCCAG 2 11515

AGGCCAG 2 11393

CACCCAG 2 11646

AGCACAG 2 11822

CCCACAG 3 11147

ATGGAAG 2 12972

CCGGAAG 2 11065

CCAGAAG 2 11512

CCCAAAG 2 11155

CACAAAG 2 11249

ACAGTTC 2 11472

AGCTGTC 2 11295

CCTGGTC 2 11421

AAAAGTC 2 11619

CTTTCTC 2 11934

CCTTCTC 2 12750

CTGGCTC 2 11672

CCGGCTC 2 11519

CATCCTC 3 12164

AGGCCTC 2 12613

GAGCCTC 2 12958

CAGCCTC 3 13012

GACCCTC 3 12690

CTCACTC 2 12931

CAGGATC 2 12245

AGCCATC 2 11060

CGTGTGC 2 12097

CTGGTGC 2 11098

CCTCTGC 3 12393

AGGCTGC 2 12936

GAGCTGC 3 12869

CAGCTGC 2 12570

CTCCTGC 2 11908

CTGATGC 2 11847

ATCATGC 2 12143

CACATGC 2 13133

CAGTGGC 2 12492

CCCTGGC 4 12406

CCTGGGC 2 11452

ACTGGGC 2 12461

ACGGGGC 2 12529

GAGGGGC 2 12941

CAGGGGC 3 12648

CTCGGGC 2 11717

GCAGGGC 3 12720

CCAGGGC 4 12201

GAAGGGC 2 13122

AGCCGGC 2 11518

ACCCGGC 2 12995

GAGAGGC 2 13014

CCCAGGC 2 11451

CACAGGC 2 11215

CTGGCGC 2 12195

CCGGCGC 2 12033

CACACGC 2 12096

CCTGAGC 3 11669

GAGGAGC 2 12080

CATCAGC 2 12545

AGGCAGC 3 12712

GAGCAGC 2 12871

GCCCAGC 2 12573

CCCCAGC 2 12155

CCACAGC 2 13070

CAGTTCC 2 11471

CCCTTCC 2 11160

CCATTCC 2 12028

GCTGTCC 2 11296

AGGGTCC 2 12621

CCTCTCC 2 12083

CAGCTCC 2 11092

GACCTCC 2 11305

AGGATCC 2 12246

AGCTGCC 2 12569

CCCTGCC 3 12719

ACATGCC 2 13132

CTTGGCC 2 12838

CCTGGCC 2 11394

AATGGCC 2 12525

CTGGGCC 2 12574

CGGGGCC 2 12841

AGGGGCC 2 12649

CCGGGCC 2 13034

CAGGGCC 3 12982

CCCGGCC 4 12070

AGAGGCC 2 12395

CCAGGCC 3 13080

AAAGGCC 2 11391

GCGCGCC 2 12035

GGCCGCC 2 11105

CTGAGCC 2 11670

AGGAGCC 3 12512

CAGAGCC 2 12205

GCCAGCC 2 12193

CACAGCC 3 13071

GGAAGCC 2 12970

CCTTCCC 4 12353

AAGTCCC 2 11070

AGCTCCC 2 11608

GCTGCCC 3 12568

ATGGCCC 2 13032

CGGGCCC 2 11440

AGGGCCC 3 12663

CCGGCCC 2 12071

GAGGCCC 3 12831

CAGGCCC 4 12576

CTCGCCC 3 12362

GCCGCCC 2 11104

CCAGCCC 3 12781

ACAGCCC 2 11839

CAAGCCC 2 12592

CGGCCCC 2 12992

AGGCCCC 2 12650

CAGCCCC 2 11838

GGCCCCC 2 11164

AGCCCCC 2 11704

CAGACCC 2 12692

CTGGACC 2 12369

CAGGACC 2 12058

GCAGACC 2 12302

CTGCACC 3 12890

GGGCACC 2 13119

AGCCACC 2 13098

CAGAACC 2 12770

GCCCTAC 2 12289

GGGTGAC 2 11644

GGATGAC 2 12150

CCTGGAC 4 11017

GGAGGAC 2 12633

CGGAGAC 2 11590

ACCAGAC 2 12694

ACTTCAC 2 13058

CCCTCAC 2 11551

GCTGCAC 2 11095

CCTGCAC 3 12891

CAGGCAC 2 11213

AAGGCAC 2 12853

CGTCCAC 2 12792

GGGCCAC 2 12486

GAGCCAC 2 12510

CAGCCAC 2 13099

GTCCCAC 2 11678

CTCCCAC 2 13086

GCCCCAC 2 12990

CACCAAC 2 13062

GGGAAAC 2 11986

GGAAAAC 2 12817

GGCGTGA 2 11544

GAGCTGA 2 11606

AAACTGA 2 11409

AGGATGA 2 12163

CAGTGGA 2 11006

AAGTGGA 2 11433

GGCTGGA 2 12402

GCCTGGA 2 12656

CCCTGGA 4 12885

ACCTGGA 2 11016

GGTGGGA 3 13085

CCTGGGA 2 12821

CTGGGGA 2 12154

GCCGGGA 2 11076

TCAGGGA 2 12755

CCAGGGA 2 11301

TCCCGGA 2 11033

GGGAGGA 2 12634

AGGAGGA 2 11748

GAGAGGA 2 12166

AGAAGGA 2 11510

ACAAGGA 3 12519

AGGGCGA 2 12361

GGCACGA 2 11211

CTCTAGA 2 11992

GCGGAGA 2 11589

AACGAGA 2 12329

CTGCAGA 3 12773

GGCCAGA 2 11514

CACCAGA 2 12695

GCACAGA 2 11194

GGGAAGA 2 12239

AGAAAGA 2 11933

ACAAAGA 2 11248

GACTTCA 2 13057

GATGTCA 2 12147

AGCGTCA 2 11201

CCTCTCA 2 12167

AGCCTCA 2 12159

GCCCTCA 2 11552

ACCCTCA 2 11317

GAACTCA 2 13104

GACATCA 2 12146

ATTTGCA 2 12906

GCCTGCA 3 12978

ACCTGCA 2 12892

CTGGGCA 2 11177

AGGGGCA 2 11398

CCAGGCA 2 12657

ACAGGCA 3 11854

GAAGGCA 2 13047

CTGAGCA 2 12948

ATCAGCA 2 11845

GGCAGCA 2 11851

GTTTCCA 2 11859

AGTTCCA 2 11470

GCTTCCA 2 12971

GGGTCCA 2 13039

ACGTCCA 2 11001

GCCTCCA 2 11478

CCTGCCA 2 12251

GGGGCCA 2 12840

CGGGCCA 2 13033

CCGGCCA 3 12336

GGCGCCA 2 12196

GGAGCCA 3 12511

TCAGCCA 2 12048

CCAGCCA 2 12192

CTTCCCA 4 12352

CCTCCCA 3 13087

TGGCCCA 2 12485

AGGCCCA 2 12575

ACGCCCA 2 12459

GGCCCCA 2 11289

AGCCCCA 2 12967

GCCCCCA 2 11138

AGGACCA 2 12057

TGCACCA 2 11085

GCCACCA 2 13097

CTGGACA 2 13023

GAGGACA 2 12632

CTGCACA 2 12109

AGGCACA 2 11489

CAGCACA 2 11650

CTCCACA 2 12859

CCCCACA 3 13068

GGAAACA 2 11858

AGTGGAA 2 11913

GCTGGAA 2 11468

GATGGAA 2 12542

CTGGGAA 2 12820

CGGGGAA 2 13051

GAGGGAA 2 11261

CCCGGAA 2 11066

AGAGGAA 2 12478

TGCAGAA 2 12772

GCCAGAA 2 11513

GGAAGAA 2 12539

GGGCCAA 2 12839

TTCCCAA 2 12351

CCCCCAA 2 12801

TCACCAA 2 13061

TGGGAAA 2 11985

AAAGAAA 2 11931

CCTCAAA 2 12445

TTGCAAA 2 12907

TCCCAAA 2 11156

GGGAAAA 2 12818

AAGAAAA 2 11930
