# Supplementary figures and images for: EDIR: exome database of interspersed repeats
Source: Bioinformatics. 2022 Dec 1;39(1):btac771. doi: 10.1093/bioinformatics/btac771 (PMC9805566; doi:10.1093/bioinformatics/btac771)

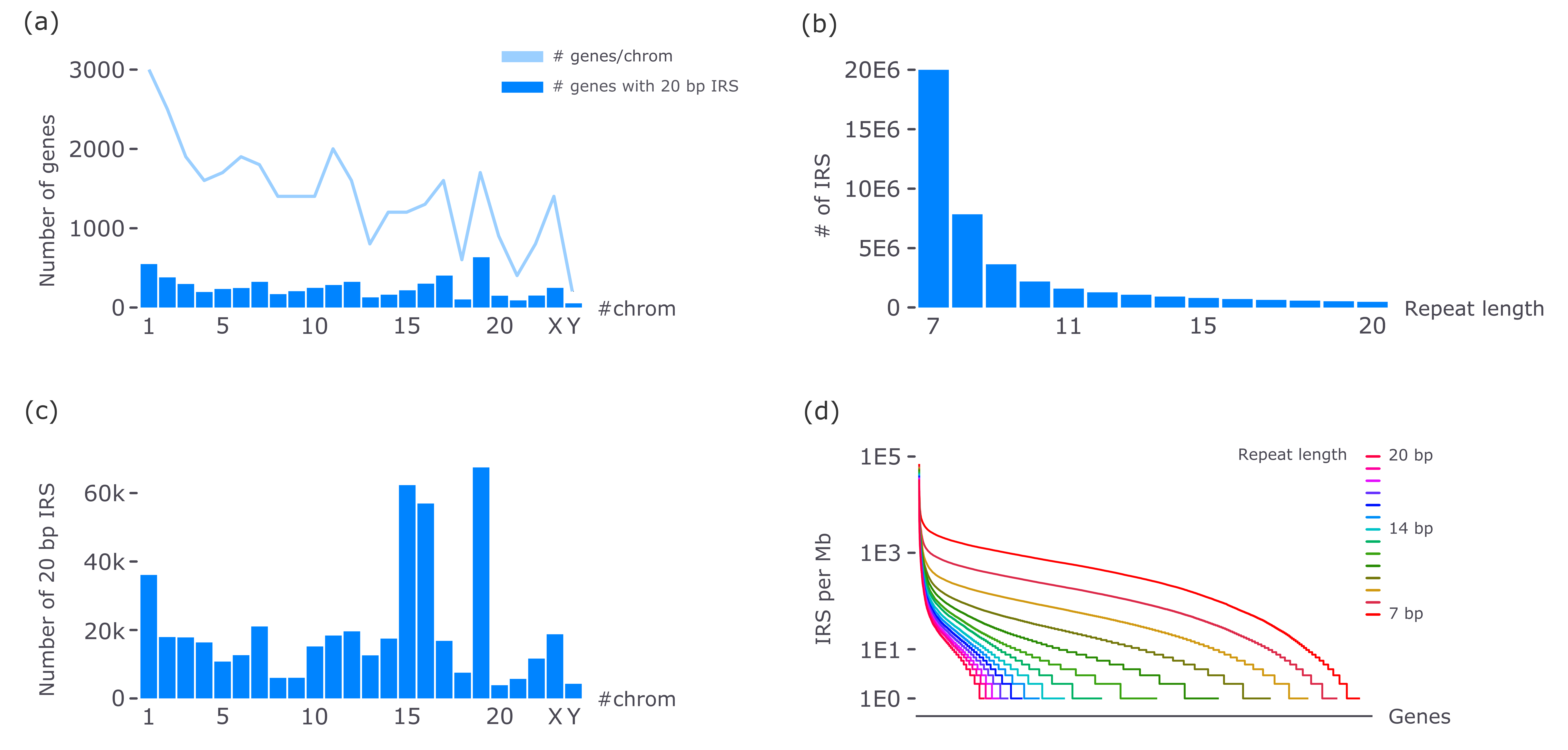

Supplement: btac771_Supplementary_Data [file btac771_supplementary_data.zip › btac771_Supplementary_Data/Supplementary_Figure_1.tif]
